# The effects of obesity on skeletal muscle regeneration

**DOI:** 10.3389/fphys.2013.00371

**Published:** 2013-12-17

**Authors:** Dmitry Akhmedov, Rebecca Berdeaux

**Affiliations:** Department of Integrative Biology and Pharmacology and Graduate School of Biomedical Sciences, University of Texas Health Science Center at HoustonHouston, TX, USA

**Keywords:** obesity, type 2 diabetes, lipids, skeletal muscle, muscle regeneration, satellite cells, leptin, lipotoxicity

## Abstract

Obesity and metabolic disorders such as type 2 diabetes mellitus are accompanied by increased lipid deposition in adipose and non-adipose tissues including liver, pancreas, heart and skeletal muscle. Recent publications report impaired regenerative capacity of skeletal muscle following injury in obese mice. Although muscle regeneration has not been thoroughly studied in obese and type 2 diabetic humans and mechanisms leading to decreased muscle regeneration in obesity remain elusive, the initial findings point to the possibility that muscle satellite cell function is compromised under conditions of lipid overload. Elevated toxic lipid metabolites and increased pro-inflammatory cytokines as well as insulin and leptin resistance that occur in obese animals may contribute to decreased regenerative capacity of skeletal muscle. In addition, obesity-associated alterations in the metabolic state of skeletal muscle fibers and satellite cells may directly impair the potential for satellite cell-mediated repair. Here we discuss recent studies that expand our understanding of how obesity negatively impacts skeletal muscle maintenance and regeneration.

Obesity and associated disorders are quickly reaching a global epidemic scale. Over 500 million people worldwide are overweight or obese (World Health Organization, 2013). Obesity is highly associated with development of metabolic syndrome, type 2 diabetes, non-alcoholic fatty liver disease (NAFLD) and cardiovascular disorders (Kahn et al., 2006; Lavie et al., 2009; Samuel and Shulman, 2012). In obese individuals, lipids excessively accumulate in adipose tissues and ectopically accumulate in non-adipose tissues including skeletal muscle (Unger et al., 2010). Lipids in skeletal muscle have been extensively studied in the context of insulin sensitivity. However, lipid overload in muscle appears to affect not only insulin signaling, but also muscle maintenance and regeneration. The underlying mechanisms are not fully understood, but recent experimental data suggest that multiple factors such as accumulation of toxic lipid metabolites and low-grade inflammation result in impaired muscle regeneration under conditions of obesity. The impact of obesity on skeletal muscle maintenance and physiology has been addressed in rodent models of obesity, including leptin-deficient *Lep^ob/ob^* mice (commonly termed “*ob/ob*”), leptin receptor-deficient *Lepr^db/db^* mice (termed “*db/db*”) and obese Zucker rats (which also have a leptin receptor mutation) (Kurtz et al., 1989; Tschop and Heiman, 2001), as well as in mice and rats fed a high-fat diet. All of these animals have increased whole body lipid content and develop hyperglycemia and insulin resistance, a phenotype similar to type 2 diabetes (reviewed in Unger, 2003).

Here we will discuss the sources of lipids that directly affect skeletal muscle, review studies investigating muscle regeneration in obesity models, and discuss possible mechanisms underlying impaired regenerative capacity of skeletal muscle in obese animals (summarized in Figure 1).

**Figure 1:**
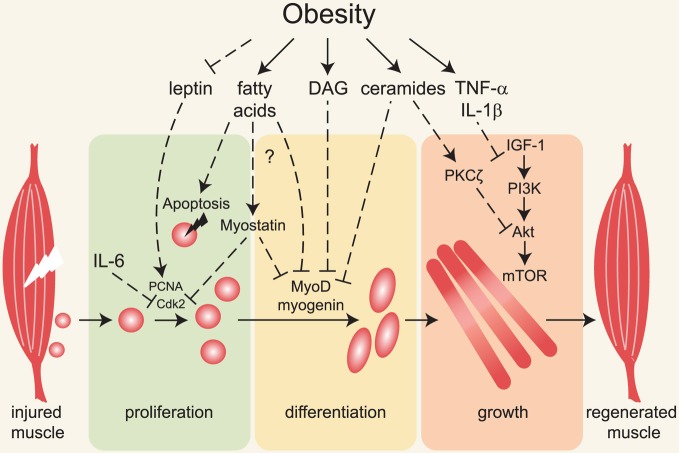
**Major mechanisms linking obesity with impaired muscle regeneration.** Obesity is associated with insulin and leptin resistance, elevated circulating and intramuscular fatty acids, diacylglycerols, ceramides and pro-inflammatory cytokines. Following muscle injury, satellite cells (depicted adjacent to muscle on left) are activated, proliferate, differentiate and form myofibers that grow and replace damaged tissue. Impairment of these processes underlies inefficient muscle regeneration in obese rodents. Defective leptin signaling can contribute to decreased satellite cell proliferation and impaired muscle hypertrophy, but the molecular mechanisms are not known. Fatty acids, diacylglycerols (DAG) and ceramides induce apoptosis and decrease myoblast proliferation and differentiation, possibly via activation of myostatin and inhibition of MyoD and myogenin expression and/or activity. Ceramides and pro-inflammatory cytokines inhibit muscle growth in part by inhibiting the IGF-1/Akt /mTOR pathway.

## Obesity and skeletal muscle lipid accumulation

Obesity is characterized by elevated adipose storage in subcutaneous and visceral adipose depots and non-adipose organs, a phenomenon called ectopic lipid accumulation (Van Herpen and Schrauwen-Hinderling, 2008). In addition, obese individuals have increased circulating fatty acids (Boden and Shulman, 2002; Mittendorfer et al., 2009) and high ectopic lipid deposition in skeletal muscle partially resulting from increased fatty acid uptake from the circulation (Goodpaster et al., 2000b; Sinha et al., 2002; Bonen et al., 2004; reviewed in Goodpaster and Wolf, 2004). Lipids within skeletal muscle are comprised of two pools: extramyocellular lipids (EMCL) localized in adipose cells between myofibers and intramyocellular lipids (IMCL) located within muscle cells (Sinha et al., 2002; Boesch et al., 2006). A portion of EMCL comprises adipose tissue closely associated with the muscle, referred to as intermuscular adipose tissue (IMAT) (Goodpaster et al., 2000a). Although IMAT accumulation in obese patients is positively correlated with insulin resistance and reduced muscle performance (Goodpaster et al., 2000a; Hilton et al., 2008), this adipose depot does not appear to affect muscle mass (Lee et al., 2012a), and its effects on muscle regeneration have not been addressed. IMCL are comprised of neutral lipids triacylglycerols (TAG) and cholesterol esters, mainly localized to lipid droplets (reviewed in Fujimoto et al., 2008; Thiele and Spandl, 2008) as well as lipid metabolites, such as long-chain acyl CoAs, diacylglycerols and ceramides. Elevated TAG content and increased numbers of lipid droplets have been observed in muscle biopsies from obese people (Simoneau et al., 1995; Malenfant et al., 2001). Genetically obese mice (*ob/ob* and *db/db*) and obese Zucker rats also have increased IMCL (Kuhlmann et al., 2003; Unger, 2003; Fissoune et al., 2009; Ye et al., 2011). Long-chain acyl CoAs, diacylglycerols and ceramides accumulate in skeletal muscles of obese humans, *ob/ob* and *db/db* mice and obese Zucker rats (Turinsky et al., 1990; Hulver et al., 2003; Adams et al., 2004; Holland et al., 2007; Magnusson et al., 2008; Lee et al., 2013; Turner et al., 2013) and negatively affect cell signaling and metabolism; the defects are collectively referred to as lipotoxicity (Lelliott and Vidal-Puig, 2004; Kusminski et al., 2009). In skeletal muscle, lipotoxic species interfere with insulin signaling and are thought to be partly responsible for insulin resistance in obesity (reviewed in Timmers et al., 2008; Bosma et al., 2012; Coen and Goodpaster, 2012). However, it remains largely unknown what other physiologic processes are impaired by these lipid metabolites in skeletal muscle. In the following sections we will focus on recent findings on how obesity, and in some cases lipids, impair muscle progenitor cell function and muscle regeneration and regrowth.

## Effects of obesity on muscle progenitor cells

Insulin resistance and mitochondrial and metabolic dysfunction are perhaps the most prominent muscle abnormalities that negatively impact whole body metabolism and physical performance in states of obesity and type 2 diabetes. Skeletal muscle maintenance depends on ongoing repair, regeneration and growth, all of which decline during aging (reviewed in Jang et al., 2011). Obesity rates increase with aging, which is also accompanied by reduced regenerative capacity and muscle strength. Thus, as average life span increases, it is of growing clinical importance to understand whether obesity impacts muscle maintenance and regeneration and to identify mechanisms that may be targeted for therapeutic benefit.

Skeletal muscle regeneration after injury requires the activity of muscle stem cells and satellite cells, which remain associated with skeletal myofibers after development (reviewed in Wang and Rudnicki, 2012). Muscle regeneration is commonly experimentally induced by intramuscular injection of a myotoxic agent, such as cardiotoxin, notexin or barium chloride. Freeze-induced injury is an alternative model of muscle injury entailing application of steel cooled to the temperature of dry ice to the muscle (Warren et al., 2007). In normal animals, these injuries cause local myofiber necrosis and inflammation, followed by satellite cell activation, proliferation, differentiation, fusion and ultimately regrowth of myofibers to approximately the same size as the original within about three weeks (Figure 1 and Charge and Rudnicki, 2004). Satellite cells are required for regenerative myogenesis (Lepper et al., 2011; Gunther et al., 2013). Currently there is a controversy regarding requirement of satellite cells for skeletal muscle hypertrophy. Load-induced hypertrophy in humans and rodents is accompanied by satellite cell activation, proliferation and fusion with existing myofibers (Rosenblatt et al., 1994; Kadi et al., 2004; Petrella et al., 2008; Bruusgaard et al., 2010). However, genetic ablation studies in mice demonstrated that satellite cells do not appear to be required for hypertrophy induced by mechanical overload (McCarthy et al., 2011; Jackson et al., 2012; Lee et al., 2012b). Although efficient hypertrophy in rodents does not strictly require satellite cell fusion to myofibers, nuclear accretion due to satellite cell fusion is thought to promote hypertrophy by supporting the growing cytoplasm. In addition, muscle regenerative capacity declines with aging, and this is thought to be due in part to reduced satellite cell function (reviewed in Jang et al., 2011). Thus, although it is still not settled to what extent this specific progenitor population is required for maintenance of adult muscle, it is clear that identification of therapeutic targets to stimulate and maintain activity of these cells has potential to improve metabolism and strength in aging and obese humans. Recent data indicate that skeletal muscle regeneration is significantly impaired in models of diabetes and obesity, possibly due to impaired muscle progenitor cell function.

### Lipotoxicity in myoblasts

Several groups have modeled lipid overload by incubating cultured muscle cells with fatty acids or lipid metabolites. During differentiation of L6 myoblasts, exogenous ceramides markedly reduce expression of the myogenic transcription factor myogenin, likely via inhibition of phospholipase D, while inhibitors of ceramide synthesis potentiate myogenin expression and accelerate myotube formation (Mebarek et al., 2007). In addition, several studies showed that increasing ceramide pools either by palmitate loading or silencing of stearoyl-CoA desaturase 1 (SCD1), which normally desaturates fatty acids and reduces the pool of saturated fatty acids that are converted to ceramides, results in increased apoptosis in differentiated L6 and C2C12 muscle cells (Turpin et al., 2006; Rachek et al., 2007; Peterson et al., 2008b; Henique et al., 2010; Yuzefovych et al., 2010). These findings suggest that the elevated fatty acids in obesity could directly harm the muscle fibers and satellite cells.

To test the effect of intracellular free fatty acid accumulation on myoblast viability and myogenesis, Tamilarasan, et al. used C2C12 cells stably transfected with human lipoprotein lipase (LPL), which converts TAGs to free fatty acids and glycerol (Tamilarasan et al., 2012). In spite of an approximately tenfold increase in intracellular free fatty acids and TAGs, cell viability and proliferation were similar to control cells. However, LPL-expressing cells showed defective differentiation accompanied by markedly decreased expression of *MyoD*, *myogenin*, and myosin heavy chain as well as a reduced number of myotubes (Tamilarasan et al., 2012). In mice, acute triglyceride infusion resulted in increased plasma free fatty acid and diacylglycerol levels and increased caspase-3 activity in gastrocnemius muscle (Turpin et al., 2009). However, in the same study, *ob/ob* mice and mice fed high-fat diet for 12 weeks did not show increased apoptosis, autophagy or proteolysis in muscle despite elevated plasma free fatty acids, muscle diacylglycerols and ceramides (Turpin et al., 2009). In contrast with this result, another group observed increased caspase-3 activation in gastrocnemius muscle in mice after 16 weeks of high-fat diet feeding (Bonnard et al., 2008), probably secondary to elevated reactive oxygen species (ROS), oxidative stress and mitochondrial dysfunction. Because cell viability and apoptosis were not directly assessed in this study, it is difficult to conclude if caspase-3 activation was accompanied by increased apoptosis (Bonnard et al., 2008). It is possible that pro-apoptotic effects of caspase-3 in muscle from obese animals are counteracted by increased expression of pro-survival Bcl2 and transcriptional downregulation of other pro-apoptotic genes, such as *caspase8, caspase14*, *Fadd*, and multiple genes involved in TNF-α signaling (Turpin et al., 2009)*.* Therefore, although fatty acids and ceramides induce apoptosis in muscle cells *in vitro*, it appears that elevated lipid metabolites do not impair muscle cell viability *in vivo*. *In vitro* studies have raised the interesting possibility that fatty acids and possibly other lipid metabolites interfere with the myogenic differentiation program, suggesting that perhaps differentiation during muscle regeneration would be impaired in obese animals.

### Muscle regeneration in obesity models

Several recent studies have employed myotoxins and freeze injury to evaluate muscle regeneration in obese or diabetic mice. In mice fed high-fat diet for 8 months, Hu, et al. observed reduced tibialis anterior (TA) muscle mass after cardiotoxin injury, associated with smaller myofibers, larger interstitial spaces and increased collagen deposition compared with lean mice (Hu et al., 2010). Similarly, a short period of high-fat diet (3 weeks) in young mice (aged 3–6 weeks) resulted in reduced numbers of satellite cells and impaired regeneration of TA muscle after cold-induced injury (Woo et al., 2011). A similar effect on satellite cell number and regeneration was observed in young mice with prenatal malnutrition, which also results in elevated adiposity (Woo et al., 2011). Although proliferation rates were not directly assessed in this study, the data collectively suggest that high adiposity depresses proliferative capacity of satellite cells either due to intrinsic metabolic properties of the muscle or satellite cells or alterations of circulating metabolites after high-fat feeding. However, in other studies, intermediate durations (12 weeks) of high fat feeding did not markedly impair the size of regenerating fibers of extensor digitorum longus (EDL) muscle after cardiotoxin injury (Nguyen et al., 2011). Collagen deposition was not evaluated, but there do appear to be larger interstitial spaces in histological sections of regenerating muscle from the 12 week high-fat diet-fed animals (Nguyen et al., 2011) consistent with the findings of Hu et al. (2010). It is notable when comparing these studies that Hu, et al. and Woo, et al. evaluated regeneration of TA muscle while Nguyen, et al. analyzed EDL muscle. While both muscle groups are comprised of predominantly fast-twitch IIB/X fiber types, TA contains a larger proportion of oxidative type IIA fibers (Bloemberg and Quadrilatero, 2012). The choice of muscle group is an important consideration, as slow twitch muscles contain higher numbers of satellite cells per fiber (Gibson and Schultz, 1983). Thus, effects of high-fat diet feeding on different functional aspects of muscle regeneration may depend on the muscle studied and the type of analysis performed. Ultimate conclusions will depend on additional analyses of multiple parameters of muscle regeneration in high-fat diet fed animals, including careful analysis of proliferation, muscle progenitor number, as well as resolution of inflammation, fibrosis and fiber caliber during regrowth.

Effects of lipid overload on skeletal muscle regeneration have specifically been assessed in transgenic mice overexpressing LPL in skeletal muscle (Levak-Frank et al., 1995; Tamilarasan et al., 2012). Overexpression of LPL in muscle results in an approximately eightfold increase in LPL activity, increased free fatty acid uptake and three- to fourfold increases in free fatty acid and TAG concentrations in gastrocnemius muscle. By two months of age, transgenic mice develop severe myopathy, which is detected histologically as regenerating myofibers with centrally localized nuclei, in addition to perturbed sarcomere structure, excessive glycogen storage, increased protein degradation and apoptotic nuclei (Levak-Frank et al., 1995; Tamilarasan et al., 2012). Ten days after cardiotoxin injury, myofiber cross-sectional area in LPL-transgenic mice is reduced compared to wild-type mice, indicating that intracellular lipid accumulation impairs muscle regeneration (Tamilarasan et al., 2012), either directly or indirectly. The defect in regeneration might result from reduced differentiation of progenitor cells, as LPL overexpression blocks myogenic differentiation of C2C12 cells (Tamilarasan et al., 2012) as described above. This, however, has not yet been tested. The pronounced muscle degenerative phenotype in LPL-expressing mice is most likely explained by lipotoxicity caused by the several-fold increase in intracellular free fatty acid and TAG concentrations. In comparison, high-fat diet feeding usually results in a 30–50% increase in intramuscular TAG in rodents (Marotta et al., 2004; Bruce et al., 2009; Ussher et al., 2010). The ultimate extent of lipotoxicity in skeletal muscle *in vivo* will therefore likely depend on the extent of lipid infiltration.

### Leptin signaling

In genetically obese *ob/ob* and *db/db* mice, which have more severe insulin resistance than high-fat diet-fed mice, EDL myofiber regeneration after cardiotoxin injury is blunted (Nguyen et al., 2011). This finding could suggest that leptin signaling is important for skeletal muscle regeneration. In support of this model, injury-induced satellite cell proliferation is specifically impaired in leptin signaling-deficient mouse models, but not in the two high-fat diet models (Hu et al., 2010; Nguyen et al., 2011). Notably, *ob/ob* and *db/db* mice show defects of early regeneration stages: decreased proliferation and reduced MyoD expression are most evident at day 5 post-injury (Nguyen et al., 2011). In agreement with this result, basal rates of satellite cell proliferation are reduced in both mice and obese rats with leptin signaling deficiencies (Purchas et al., 1985; Peterson et al., 2008a), suggesting reduced proliferative capacity. Recombinant leptin stimulates proliferation and *MyoD* and *myogenin* expression in myoblasts from wild-type mice, but myoblasts from mice lacking all forms of the leptin receptor (referred to as POUND mice) show decreased expression of *MyoD* and *myogenin* transcripts and decreased myotube formation during differentiation *ex vivo* (Arounleut et al., 2013). Moreover, administration of recombinant leptin to *ob/ob* mice restores expression of the proliferation markers proliferating cell nuclear antigen (PCNA) and cyclin D1, which may account for the muscle growth-promoting effect of recombinant leptin in leptin-deficient animals (Sainz et al., 2009). In C2C12 myoblasts, leptin also stimulates proliferation but does not appear to promote MyoD or myogenin expression or differentiation (Pijet et al., 2013). Although leptin clearly has stimulatory effects on mouse myoblasts and muscle, it is not clear whether leptin promotes myoblast proliferation in all species. Leptin receptors are poorly abundant in porcine muscle, and recombinant leptin has no effect on proliferation of primary porcine myoblasts cultured in serum free medium or on protein accretion as these cells differentiated (Will et al., 2012). In line with this finding, lean and obese leptin receptor-deficient Zucker rats exhibit comparable BrdU incorporation, expression of myogenic regulatory factors, activation of pro-hypertrophic signaling pathways and gain of muscle mass in response to overload, demonstrating that leptin signaling *per se* is not required for satellite cell activation and muscle hypertrophy, at least in rats (Peterson et al., 2008a).

In addition to the activity of satellite cells, macrophages also contribute to regeneration of injured muscle by facilitating removal of tissue debris (Arnold et al., 2007). Leptin stimulates proliferation and activation of macrophages (Santos-Alvarez et al., 1999; Raso et al., 2002), pointing to another possible mechanism by which leptin resistance could impair muscle regeneration. Nguyen, et al. provided data supporting this hypothesis: in injured muscle of *ob/ob* and *db/db* mice, macrophage accumulation is decreased during early regeneration (Nguyen et al., 2011). In addition, these authors observed markedly decreased angiogenesis after injury in *ob/ob* and *db/db* mice (Nguyen et al., 2011). The data suggest that leptin could potentiate muscle regeneration by regulating macrophage activity and/or by stimulating vascularization. Vascularization potentiates regrowth of regenerating muscle in mice (Ochoa et al., 2007; Deasy et al., 2009). It appears that vascularization is not only important for nutrient availability but also myofiber growth. Vascular endothelial growth factor (VEGF), elevated during angiogenesis, promotes regeneration by directly stimulating myofiber growth (Arsic et al., 2004; Messina et al., 2007). As leptin resistance is often observed in obese and type 2 diabetic humans (Maffei et al., 1995; reviewed in Martin et al., 2008) it is possible that lack of leptin signaling could contribute to poor vascularity and compromised satellite cell function.

### Inflammation

In skeletal muscle, inflammation is activated after injury and is coordinated with myogenic differentiation to achieve efficient muscle regeneration (reviewed in Mann et al., 2011; Kharraz et al., 2013). Immediately after muscle injury, an acute inflammatory stage ensues characterized by infiltration of pro-inflammatory M1 macrophages that remove tissue debris. Later, a different population of macrophages (M2) resolves inflammation. Accumulating data show that macrophages not only mediate inflammation but also support satellite cells during skeletal muscle regeneration. In mice, deletion of chemokine receptor-2 (CCR-2) impairs macrophage infiltration after muscle injury and results in inefficient muscle regeneration (Warren et al., 2005). In co-culture experiments *in vitro*, macrophages stimulate satellite cell proliferation (Cantini et al., 1994; Massimino et al., 1997; Merly et al., 1999). When transplanted together with satellite cells into muscle of *Dmd^mdx^* mice, a mouse model of human Duchenne muscular dystrophy, macrophages stimulate satellite cell survival and proliferation (Lesault et al., 2012). This potentiation effect is likely mediated, at least in part, by pro-inflammatory cytokines TNF-α and IL-6, which promote myoblast proliferation and migration *in vitro* (Li, 2003; Torrente et al., 2003; Wang et al., 2008; Toth et al., 2011). However, TNF-α and another pro-inflammatory cytokine IL-1α also prevent myogenic differentiation (Miller et al., 1988; Layne and Farmer, 1999; Langen et al., 2001; Trendelenburg et al., 2012). During later stages of regeneration, TGF-β and IL-10 secreted by anti-inflammatory M2 macrophages promote myogenic differentiation (Arnold et al., 2007; Deng et al., 2012). Thus, the interplay between macrophages and satellite cells is precisely temporally orchestrated during skeletal muscle regeneration.

Obesity is recognized as a state of chronic inflammation with increased circulating pro-inflammatory cytokines TNF-α, IL-1β and IL-6 (reviewed in Wellen and Hotamisligil, 2005; Gregor and Hotamisligil, 2011). The effects of chronically elevated cytokines on satellite cell maintenance, activation and proliferation are not well understood, but it appears that chronic exposure to cytokines has distinct effects on myoblast proliferation and differentiation from acute exposure. For example, in a mouse model of chronic inflammation in which TNF-α is constitutively expressed in lung and becomes chronically elevated in the circulation, skeletal muscle becomes atrophic and myoblast proliferation and differentiation are reduced in response to mechanical loading (Langen et al., 2006). Similarly, chronic, local delivery of IL-6 to muscle of young rats inhibits muscle growth and stimulates expression of cyclin-dependent kinase inhibitor *p21*, suggesting decreased satellite cell proliferation, although this has not been tested (Bodell et al., 2009). It is possible that in chronic inflammation the normal coordination between macrophages and muscle satellite cells is impaired and contributes to impaired satellite cell function. It would be interesting to manipulate cytokine signaling in obesity models to determine whether the chronic inflammation that accompanies obesity in fact does impair muscle satellite cell proliferation and differentiation and ultimately muscle growth.

### Myostatin

Myostatin is a member of the TGF-β family of secreted proteins known to prevent muscle regeneration and growth (reviewed in Joulia-Ekaza and Cabello, 2006; Kollias and McDermott, 2008). Interestingly, myostatin expression is increased in skeletal muscle of extremely obese women (Hittel et al., 2009) and of *ob/ob* and high-fat diet-fed mice (Allen et al., 2008). In C2C12 myoblasts, recombinant or overexpressed myostatin decreases proliferation most likely by stimulating expression of the cyclin-dependent kinase inhibitor p21, resulting in inhibition of Cdk2 and impaired G1 to S phase transition (Thomas et al., 2000; Taylor et al., 2001; Joulia et al., 2003). Moreover, proliferation of satellite cells in *myostatin*-null mice is markedly increased (McCroskery et al., 2003). Myostatin also represses transcription of myogenic regulatory factors through direct activation of Smad2/3 proteins, which repress expression of *MyoD* and *myogenin*. In addition, Smad3 represses MyoD activity through direct interaction (Liu et al., 2001; Langley et al., 2002). Elevated myostatin in obese people correlates with increased phosphorylation of Smad2/3 proteins and an approximately two-fold decrease in *MyoD* and *myogenin* transcript levels (Watts et al., 2013). Thus, increased myostatin in obese animals may contribute to defects in regeneration and maintenance of muscle mass (Figure 1).

The source and mechanism by which myostatin becomes elevated in obese subjects remain obscure. Expression of the *myostatin* gene is stimulated in myocytes by several pathways including glucocorticoid signaling (Salehian et al., 2006) possibly via C/EBP-δ (Allen et al., 2010) or repression of miR-27a/b (Allen and Loh, 2011). *Myostatin* expression in muscle cells has also been reported to be stimulated by FoxO1 and TGF-β/Smad3 (Allen and Unterman, 2007), MyoD (Spiller et al., 2002) and a JNK/p38-mediated signaling pathway (Han et al., 2010). It is not known which, if any, of these pathways may mediate the increase in circulating myostatin in obese patients, but it is tempting to speculate that elevated glucocorticoids commonly observed in metabolic syndrome and obesity (Anagnostis et al., 2009) could stimulate *myostatin* expression by promoter regulation (Allen et al., 2010) and modulation of miR-27a/b (Allen and Loh, 2011). Alternatively, insulin resistance may result in derepression of *myostatin* via constitutive activation of FoxO1 (Allen and Unterman, 2007); this model would be consistent with the observation of elevated myostatin in insulin resistant type 2 diabetic patients and non-obese hyperinsulinemic subjects (reviewed in Allen et al., 2011). Although skeletal muscle expresses far more *myostatin* than other tissues, it is noteworthy that *myostatin* mRNA increases by at least fifty fold in adipose tissue (primarily adipocytes) and only twofold in skeletal muscle of obese mice (Allen et al., 2008). Thus, it is possible that in obesity a large amount of myostatin could be secreted from adipose as a result of hypercortisolemia. Although myostatin is well known for its role in regulation of muscle growth, it is not clear to what extent myostatin contributes to impaired muscle regeneration observed in rodent models of obesity. Genetic manipulations disrupting myostatin signaling, such as expressing a dominant negative form of the myostatin receptor in satellite cells in an obesity model, will help to answer this question.

### Adipogenesis

Fibro/adipogenic progenitor (FAP) cells comprise a recently identified population of progenitors that reside in the muscle and become activated after muscle damage in mice (Joe et al., 2010; Heredia et al., 2013). Unlike myogenic progenitors, FAP cells do not fuse or differentiate into myofibers. Instead, FAP cells support myogenesis likely by enhancing proliferation and differentiation of myogenic progenitors through secretion of factors such as IL-6 (Joe et al., 2010). The signals that regulate FAP cell differentiation are incompletely understood. FAP cells spontaneously differentiate into adipocytes *in vitro* and when transplanted into skeletal muscle with fatty infiltration, but not when transplanted into healthy skeletal muscle (Joe et al., 2010). Using a co-culture system, Uezumi, et al. found that muscle satellite cells inhibit adipogenic differentiation of FAP cells likely by direct physical interaction (Uezumi et al., 2010), though the signal is unknown. If the same regulation occurs *in vivo*, then a decrease in satellite cell number, activity or proximity to FAP cells could result in increased adipogenic conversion of FAP cells and IMAT accumulation. Alternatively, exciting work by Heredia, et al. demonstrated that after skeletal muscle injury, eosinophil-derived anti-inflammatory cytokines IL-4/IL-13 promote FAP proliferation and inhibit their differentiation to adipocytes (Heredia et al., 2013). It is possible that under the pro-inflammatory conditions of obesity, the ability of satellite cells or eosinophils to inhibit adipogenic differentiation of FAP cells is compromised. As a result, FAP cells activated during injury could differentiate into adipocytes, contribute to increased IMAT, and occupy areas of the tissue once filled with skeletal myofibers. Indeed, it has been shown that muscle side population cells from dystrophic or injured tissue differentiate in culture to FAP cells and lose myogenic capacity (Penton et al., 2013). It is notable in this regard that in patients with Duchenne muscular dystrophy, the skeletal muscle eventually loses capacity for ongoing regeneration and myofibers are replaced by fatty infiltrate and collagen (Radley et al., 2007). It will be important for future studies to examine the action of FAP cells in obese animals and humans.

### Metabolism

Recently it has been recognized that satellite cells exhibit different intrinsic metabolic properties in states of quiescence, proliferation and differentiation (reviewed in Ryall, 2013). In the quiescent state, satellite cells have low energy demands, low oxygen consumption and low ATP production. In low nutrient conditions, elevated NAD^+^ levels stimulate the deacetylase SIRT1, which in turn promotes myoblast proliferation and prevents myogenic differentiation, in part via MyoD deacetylation (Fulco et al., 2003). Culturing mouse myoblasts in low glucose medium similarly prevents differentiation at least in part through SIRT1 activation (Fulco et al., 2008; reviewed in Ryall, 2012). It thus can be hypothesized that in low energy states, limited nutrient supply and the associated increase in SIRT1 activity would be beneficial to maintain a pool of muscle satellite cells. On the other hand, obesity and nutrient overload would be expected to provide unfavorable conditions for maintenance of quiescent satellite cells or for proliferation after acute injury.

Cerletti, et al. tested the corollary to this hypothesis by evaluating muscle satellite cell metabolism and function in mice after short-term (12 weeks) caloric restriction. They showed that short-term caloric restriction in mice increases both the number and myogenic capacity of muscle-associated satellite cells and enhances regeneration after freeze injury (Cerletti et al., 2012). Satellite cells isolated from calorie-restricted animals had higher mitochondrial content, enhanced oxidative metabolism and reduced glycolytic capacity accompanied by elevated SIRT1 expression. Muscle stem cells harvested from calorically restricted mice also displayed improved engraftment in dystrophin-deficient *Dmd^mdx^* mice that had not been previously subjected to caloric restriction (Cerletti et al., 2012). Thus, the altered cellular metabolic state of the satellite cells from a calorie-restricted animal was sufficient to confer benefits on a normal recipient. The beneficial effects of calorie restriction were not, however, limited to the satellite cells. Transplanted muscle stem cells had much higher engraftment efficiency when transplanted into healthy uninjured skeletal muscle of animals undergoing calorie restriction, possibly as a result of reduced inflammation in the muscle (Cerletti et al., 2012).

These findings strongly suggest that (1) muscle satellite cell metabolism is profoundly altered by the systemic nutritional environment and (2) the metabolic/ inflammatory state of the organism, and therefore of the mature myofibers, also affects the health or fusion capacity of satellite cells. Accumulation of SIRT1 protein in the satellite cells from calorically restricted mice could theoretically stimulate proliferation and oxidative metabolism, resulting in a larger satellite cell pool. In obesity, perturbations of intrinsic satellite cell metabolism could negatively affect the proliferation and activity of the satellite cell pool, but this exciting field is still emerging.

## Muscle regrowth after injury in obese animals

A common finding among the aforementioned *in vivo* studies of skeletal muscle regeneration in obese animals is reduced recovery of muscle mass and function after injury (Hu et al., 2010; Nguyen et al., 2011; Tamilarasan et al., 2012). This may occur secondary to reduced satellite cell function or as a result of defective hypertrophic growth after initial satellite cell differentiation and fusion. In this section, we will discuss some potential mechanisms underlying defective muscle regrowth after injury in obese animals.

### IGF-1/Akt signaling

In normal skeletal muscle, the balance between muscle hypertrophy and atrophy is largely regulated by the IGF-1/Akt signaling pathway (reviewed in Glass, 2010), which stimulates mTOR-dependent protein synthesis and inhibits FOXO-dependent transcription of muscle-specific E3 ubiquitin ligases (Bodine et al., 2001; Sartorelli and Fulco, 2004; Bodine, 2006). The balance between muscle growth and atrophy is dysregulated in obesity. In obese mice and Zucker rats, muscle growth in response to mechanical loading is reduced due to decreased activation of Akt, p70S6 kinase and mTOR (Sitnick et al., 2009; Paturi et al., 2010). Similar mechanisms might impair muscle regrowth after injury. Indeed, in high-fat diet-fed mice, Hu, et al. found that PIP_3_ levels and PI(3)-kinase activity are reduced and expression of the lipid and protein phosphatase PTEN is increased (Hu et al., 2010). These combined changes would result in decreased Akt and mTOR activity and reduced hypertrophy. *Pten* deletion in muscle is sufficient to restore Akt phosphorylation and remarkably improves muscle growth in high-fat diet-fed mice (Hu et al., 2010). These findings clearly demonstrate that dysregulated PI(3)-kinase/Akt pathway activity in muscle of obese mice not only impairs insulin signaling but also interferes with muscle growth.

On the other hand, Nguyen, et al. observed impaired muscle growth after injury in obese *ob/ob* and *db/db* and but not in high-fat diet-fed mice (Nguyen et al., 2011). Since both *ob/ob* and *db/db* mice are deficient in leptin signaling, one interpretation is that leptin signaling is necessary for normal muscle regeneration. The authors point out that leptin could promote muscle growth by activation of PI(3)-kinase and ERK1/2 pathways (Nguyen et al., 2011). Consistently, administration of recombinant leptin to mice or C2C12 myoblasts activates janus kinase 2 (JAK2), which potentiates phosphorylation of insulin receptor substrates IRS1 and IRS2, activity of PI(3)-kinase, and phosphorylation of Akt and glycogen synthase kinase 3 (GSK3) (Kellerer et al., 1997; Kim et al., 2000; Maroni et al., 2003, 2005). These studies suggest the hypothesis that leptin-dependent activation of Akt is important for regulation of muscle growth or regrowth after injury. In further support of this model, leptin treatment of *ob/ob* mice increases the mass of multiple skeletal muscle groups, including gastrocnemius, EDL and soleus, with concomitant decreased expression of muscle-specific E3 ubiquitin ligases MAFbx and MuRF1 in gastrocnemius muscle (Sainz et al., 2009).

The toxic lipid metabolites diacylglycerols and ceramides also impair IGF-1/Akt signaling. In skeletal muscle and liver, diacylglycerols activate PKCε and PKCθ, which phosphorylate multiple serine residues of insulin receptor substrate-1 (IRS-1) directly or via JNK and IKKβ ultimately leading to insulin resistance (Yu et al., 2002; Li et al., 2004; reviewed in Samuel et al., 2010; Turban and Hajduch, 2011). Interestingly, PKCθ deletion in dystrophic *Dmd^mdx^* mice increases expression of myogenin and myosin heavy chain and decreases necrotic areas in the muscle (Madaro et al., 2012). Similarly, stable PKCθ knockdown in C2C12 cells increases expression of *myogenin* and myosin heavy chain and potentiates myotube formation *in vitro* (Marino et al., 2013). The other major class of toxic lipid intermediates, ceramides, inhibits Akt by two distinct mechanisms. In C2C12 myoblasts, 3T3-L1 adipocytes and PC-12 cells, ceramides activate protein phosphatase 2A, leading to Akt dephosphorylation (Salinas et al., 2000; Cazzolli et al., 2001; Chavez et al., 2003; Stratford et al., 2004). In L6 myotubes, ceramides induce PKCζ-dependent Akt phosphorylation on Thr34, which blocks Akt translocation to the plasma membrane (Hajduch et al., 2001; Powell et al., 2003, 2004; reviewed in Bikman and Summers, 2011). In addition, ceramides impair amino acid uptake in L6 myotubes by decreasing the amount of the membrane-associated amino acid transporter SNAT2, with concomitant reduction of p70S6 kinase phosphorylation and protein synthesis (Hyde et al., 2005). All of these events would be expected to inhibit myofiber growth. It is likely that a similar mechanism contributes to impaired muscle regrowth during regeneration in obese animals (Figure 1).

### Inflammation

Pro-inflammatory cytokines TNF-α, IL-1β and IL-6 inhibit IGF-1/Akt signaling and de-repress transcription of muscle ubiquitin ligases *Mafbx* and *Murf1* and potentiate skeletal muscle atrophy (reviewed in Glass and Roubenoff, 2010). Thus, in addition to the possible negative effects on myoblast proliferation and differentiation, increased circulation of TNF-α, IL-1β and IL-6 could counteract anabolic growth of skeletal muscle during regeneration in obese animals (Figure 1). For example, treatment of human, porcine or mouse (C2C12) myoblasts with TNF-α or IL-1β prevents IGF-1-stimulated protein synthesis (Frost et al., 1997; Broussard et al., 2003, 2004). In rats, 16 weeks of high-fat diet feeding results in decreased Akt and mTOR phosphorylation and increased apoptosis that correlates with upregulation of TNF-α receptors in the muscle (Sishi et al., 2011). Interestingly, TNF-α treatment increases ceramide synthesis in C2C12 myoblasts and L6 myotubes, and exogenous ceramides cause atrophy of L6 myotubes (Strle et al., 2004; De Larichaudy et al., 2012). In support of the idea that ceramides mediate effects of TNF-α on myotubes, ceramide synthesis inhibitors block the inhibitory effect of TNF-α on IGF-1-stimulated protein synthesis (Strle et al., 2004) and prevent TNF-α induced atrophy (De Larichaudy et al., 2012). It is therefore possible that in obese animals, elevated TNF-α impairs IGF-1 signaling and muscle regrowth via ceramides and toxic lipid intermediates, which also directly inhibit satellite cell activity.

## Concluding remarks

The influence of obesity on skeletal muscle regeneration and maintenance is an emerging area that is poorly mechanistically understood. So far, this topic has been primarily addressed in studies on obese rodents. Regenerative capacity is particularly impaired by severe obesity such as in genetically obese *ob/ob* and *db/db* mice. Identifying factors that specifically block muscle regeneration in obese animals is challenging because obesity is accompanied by several abnormalities, including but not limited to ectopic accumulation of multiple lipid species, insulin and leptin resistance, chronic inflammation and metabolic disturbances (Figure 1). Using genetic models and pharmacological approaches to block synthesis of specific lipid species and modulate production and signaling of cytokines will help to determine which lipid species and cytokines specifically impair regeneration in obese animals. Another challenge is determining how obesity affects different steps during regeneration such as satellite cell activation and proliferation, myoblast differentiation, fusion and myofiber growth. In this regard, intriguing new studies linking global metabolism, cellular metabolism and satellite cell capacity for engraftment may facilitate identification of new molecular mechanisms that could be targeted therapeutically. An important open question is whether and to what extent obesity impairs muscle regeneration in humans and whether impaired muscle regeneration contributes to poor wound healing in type 2 diabetic patients (reviewed in Greenhalgh, 2003), or whether poor vascular function itself impairs satellite cell function and skeletal muscle regeneration in obese and type 2 diabetic people. In obese and type 2 diabetic patients, exercise and low calorie diet aimed at reducing lipid oversupply and stimulating metabolism could be beneficial not only by improving whole body metabolism but also perhaps by promoting anabolic growth of muscle via improved satellite cell viability and function. Stimulation or preservation of satellite cells could, in turn, enable these individuals to become stronger and more active and to possibly prevent further IMAT accumulation. In addition to abnormalities discussed here, obese and type 2 diabetic individuals suffer from complications, such as peripheral neuropathy, which we do not address directly in this review (reviewed in Vincent et al., 2011; Ylitalo et al., 2011). As innervation is required for skeletal muscle maintenance and regeneration in rodents (d'Albis et al., 1988; Rodrigues Ade and Schmalbruch, 1995; Billington, 1997), it is possible that peripheral neuropathy contributes to impaired skeletal muscle regeneration in obese and type 2 diabetic humans and could prevent putative salutary effects of strategies to promote satellite cell function. Ultimate conclusions about the effects of obesity on muscle regeneration await the results of the next generation of experiments that explore signaling mechanisms and more fully characterize muscle regeneration in obese rodents and humans.

### Conflict of interest statement

The authors declare that the research was conducted in the absence of any commercial or financial relationships that could be construed as a potential conflict of interest.
